# Global effect of COVID-19 pandemic on physical activity, sedentary behaviour and sleep among 3- to 5-year-old children: a longitudinal study of 14 countries

**DOI:** 10.1186/s12889-021-10852-3

**Published:** 2021-05-17

**Authors:** Anthony D. Okely, Katharina E. Kariippanon, Hongyan Guan, Ellie K. Taylor, Thomas Suesse, Penny L. Cross, Kar Hau Chong, Adang Suherman, Ali Turab, Amanda E. Staiano, Amy S. Ha, Asmaa El Hamdouchi, Aqsa Baig, Bee Koon Poh, Borja Del Pozo-Cruz, Cecilia H. S. Chan, Christine Delisle Nyström, Denise Koh, E. Kipling Webster, Himangi Lubree, Hong Kim Tang, Issad Baddou, Jesus Del Pozo-Cruz, Jyh Eiin Wong, Kuston Sultoni, Maria Nacher, Marie Löf, Mingming Cui, Mohammad Sorowar Hossain, P. W. Prasad Chathurangana, Uddhavi Kand, V. P. Pujitha Wickramasinghe, Rebecca Calleia, Shameema Ferdous, Thanh Van Kim, Xiaojuan Wang, Catherine E. Draper

**Affiliations:** 1 Early Start, School of Health and Society, Faculty of the Arts, Social Science and Humanities, University of Wollongong, Wollongong, NSW 2522 Australia; 2 Department of Early Childhood Development, Beijing Municipal Key Laboratory of Child Development and Nutriomics, Capital Institute of Pediatrics, Beijing, China; 3 Early Start, Faculty of the Arts, Social Science and Humanities, University of Wollongong, Wollongong, NSW 2522 Australia; 4 NIASRA - National Institute for Applied Statistics Research Australia, School of Mathematics and Applied Statistics, Faculty of Engineering and Information Sciences, University of Wollongong, Wollongong, NSW 2522 Australia; 5 Faculty of Sport and Health Education, Universitas Pendidikan Indonesia, Bandung, Jawa Barat Indonesia; 6 Precision Health Consultants (PHC Global), Karachi, Pakistan; 7 Pennington Biomedical Research Center, 6400 Perkins Rd Baton Rouge Louisiana, Pennington, 70808 USA; 8 Department of Sports Science and Physical Education, Faculty of Education, The Chinese University of Hong Kong, Hong Kong, China; 9 Unité Mixte de Recherche Nutrition et Alimentation, CNESTEN - Université Ibn Tofail (URAC-39), Regional Designated Center of Nutrition Associated with AFRA/IAEA, Pennington, USA; 10 Centre for Community Health Studies (ReaCH), Faculty of Health Sciences, Universiti Kebangsaan Malaysia, 50300 Kuala Lumpur, Malaysia; 11 Department of Sports Science and Clinical Biomechanics, Centre for Active and Healthy Ageing, University of Southern Denmark, Odense, Denmark; 12 Department of Biosciences and Nutrition, Karolinska Institutet, 14183 Huddinge, Sweden; 13 Centre of Community Education and Well-being, Faculty of Education, Universiti Kebangsaan Malaysia, 43600 UKM Bangi, Malaysia; 14 Institute of Public and Preventive Health, Augusta University, 1120 15th Street, Augusta, GA 30912 USA; 15 Vadu Rural Health Program, KEM Hospital Research Centre, Rasta Peth, Pune, India; 16 Department of Epidemiology, Faculty of Public Health, Pham Ngoc Thach University of Medicine, Ho Chi Minh City, Vietnam; 17 Departamento de Educación Física y Deporte, Universidad de Sevilla, Sevilla, Spain; 18 Department of Health, Medicine and Caring Sciences, Linköping University, 581 83 Linköping, Sweden; 19 Biomedical Research Foundation, Dhaka, Bangladesh; 20 Department of Paediatrics, Faculty of Medicine, University of Colombo, Colombo, Sri Lanka; 21 SAMRC/Wits Developmental Pathways for Health Research Unit, University of the Witwatersrand, Witwatersrand, South Africa

**Keywords:** 24-h movement behaviours, Low- and middle-income countries, Preschool, Outdoors, Play, Quarantine

## Abstract

**Background:**

The restrictions associated with the 2020 COVID-19 pandemic has resulted in changes to young children’s daily routines and habits. The impact on their participation in movement behaviours (physical activity, sedentary screen time and sleep) is unknown. This international longitudinal study compared young children’s movement behaviours before and during the COVID-19 pandemic.

**Methods:**

Parents of children aged 3–5 years, from 14 countries (8 low- and middle-income countries, LMICs) completed surveys to assess changes in movement behaviours and how these changes were associated with the COVID-19 pandemic. Surveys were completed in the 12 months up to March 2020 and again between May and June 2020 (at the height of restrictions). Physical activity (PA), sedentary screen time (SST) and sleep were assessed via parent survey. At Time 2, COVID-19 factors including level of restriction, environmental conditions, and parental stress were measured. Compliance with the World Health Organizations (WHO) Global guidelines for PA (180 min/day [≥60 min moderate- vigorous PA]), SST (≤1 h/day) and sleep (10-13 h/day) for children under 5 years of age, was determined.

**Results:**

Nine hundred- forty-eight parents completed the survey at both time points. Children from LMICs were more likely to meet the PA (Adjusted Odds Ratio [AdjOR] = 2.0, 95%Confidence Interval [CI] 1.0,3.8) and SST (AdjOR = 2.2, 95%CI 1.2,3.9) guidelines than their high-income country (HIC) counterparts. Children who could go outside during COVID-19 were more likely to meet all WHO Global guidelines (AdjOR = 3.3, 95%CI 1.1,9.8) than those who were not. Children of parents with higher compared to lower stress were less likely to meet all three guidelines (AdjOR = 0.5, 95%CI 0.3,0.9).

**Conclusion:**

PA and SST levels of children from LMICs have been less impacted by COVID-19 than in HICs. Ensuring children can access an outdoor space, and supporting parents’ mental health are important prerequisites for enabling pre-schoolers to practice healthy movement behaviours and meet the Global guidelines.

**Supplementary Information:**

The online version contains supplementary material available at 10.1186/s12889-021-10852-3.

## Introduction

With the emergence of the novel Coronavirus Disease (COVID-19) in 2019 and subsequent global pandemic declared by the World Health Organization (WHO) in March 2020, governments implemented strategies to prevent the spread of the virus and protect their citizens. In most nations, physical distancing measures and requirements to remain at home placed unprecedented restrictions on children’s ability to be active. While these measures were essential to protect the public’s health, some unintended consequences may have resulted from these restrictions [1].

Throughout a typical day, young children’s movement includes sleep, sedentary time and light- to vigorous-intensity physical activity, the latter mostly in the form of play, collectively referred to as 24-h movement behaviours. In 2019, the WHO released Global guidelines for these movement behaviours for children under 5 years of age [2]. The guidelines recommend that, during a 24-h day, pre-school children (aged 3–4 years) accumulate at least 180 min (min) physical activity (PA) – of which at least 60 min should be at moderate- to vigorous-intensity (MVPA), engage in no more than 1 h sedentary screen time (e.g. television viewing, using a computer or tablets/smartphones while sitting) (SST), and have 10–13 h good-quality sleep per day [2] Appropriate levels of movement behaviours reduce the risk of obesity and non-communicable diseases, promote health [3–5], enhance mental wellbeing [6], and are a powerful antidote to stress and prevent viral infections [7]

A significant change in young children’s lives during the COVID-19 restrictions is that they are not attending their usual places of early childhood education and care. Whether children have been able to meet movement behaviour guidelines during this time of COVID-19 restrictions is unknown, but it has been suggested that the restrictions may have considerable consequences for young children’s ability to maintain a healthy balance of movement behaviours [8].

Over a 12-month period preceding the COVID-19 pandemic, we collected data on preschool children’s movement behaviours in predominantly low- and middle-income countries (LMICs) as defined by the World Bank [9]. This provided a baseline from which to determine the impact of the pandemic on children’s movement behaviours. The aim of this study was to examine how, compared with the time period pre-COVID, the COVID-19 pandemic influenced physical activity, sedentary behaviour, screen time, and sleep among pre-schoolers. Further, the study sought to examine the relationship between COVID-related restriction levels, parent and family factors, and changes in young children’s physical activity, sedentary behaviour and sleep. We hypothesized that there would be i) increases in screen time, ii) decreases in physical activity, iii) changes in sleep patterns (bed, wake and nap times), and iv) that changes in movement behaviours would be associated with specific family and community-level COVID-19 factors.

## Methods

### Study design

Prior to the COVID-19 pandemic, 14 countries (8 LMICs) collected data between April 2019 and March 2020 as part of the SUNRISE pilot study (https://sunrise-study.com) to determine the proportion of 3- and 4-year-old children who met the WHO Global guidelines. These countries conducted follow-up data collected between May–June 2020, with participants reporting on their experience at the height of prevention and control measures in their respective countries.

The research was performed in accordance with the Declaration of Helsinki. Overall research approval for the study was obtained from the Human Research Ethics Committee (Ref: 2018/044) from the University of Wollongong, Australia. Each wave of data collection was approved by the relevant Human Research Ethics Committees in each participating country.

### Setting

Data were collected by local research teams in urban and rural settings, with participants recruited via early childhood education and care (ECEC) services and villages. At Time 1 (T1), parent survey were either conducted via interview, if there were literacy barriers, or self-administered. Follow-up (Time 2, T2) data collection was conducted via telephone interview or online survey via the Research Electronic Data Capture platform (REDCap) [10]

### Participants

To be eligible for participation at T1, children were aged ≥ 3.0 and ≤ 5.11 years. To be eligible for participation at T2, data collection at T1 needed to have occurred within the preceding 12-months.

### Variables

#### Physical activity, sedentary time and sleep

Primary parents reported their child’s total physical activity (TPA), moderate- to vigorous-intensity physical activity, sedentary screen time, and the child’s bed and wake times and nap duration, in hours and minutes per day, via survey (Table S1). Additional questions asked about screen device use before bedtime, screens in bedrooms, and sleep quality. Questions were based on the recommendations for each behaviour guideline [11] and then tested and refined as part of the SUNRISE pilot study, ensuring feasibility and acceptability among participating populations.

#### Validation of PA questions

Concurrent validity of the questions asking parents to report time spent in TPA and MVPA was evaluated using T1 data from Actigraph GT3X+ accelerometers on 436 and 377 participants, respectively (note: accelerometry data collected at T1 is not presented in this paper). Correlations were significant for TPA (r = 0.14; *P* = 0.003) and for MVPA (r = 0.16; *P* = 0.002). Classification rates for meeting or not meeting the WHO Global guidelines based on Actigraph and parent-reported data were calculated. The sensitivity and specificity were 53 and 61% for TPA and 60 and 46% for MVPA, respectively.

#### Demographics and COVID-19 factors

Child age and sex and their parents’ highest level of education were collected. Each country’s Human Development Index and World Bank income classification were recorded and the data collection locations in each country were classified as urban or rural (Table S2).

In addition, the T2 survey included questions around the circumstances families faced during COVID-19 restrictions. These included parental working arrangements, type of housing, people per household, time spent outdoors, parental stress and exhaustion levels, parental efficacy in supporting their child to maintain healthy movement behaviours, support received from their early ECEC service, and resources accessed in the home. These were adapted from a similar survey [12].

#### COVID-19 restrictions

Data on six indicators of government responses to the Coronavirus pandemic, deemed relevant to the research questions, were obtained from the Oxford COVID-19 Government Response Tracker (OxCGRT) [13]. Two indicators, *ability to go outside to exercise* and *playground closures* were added. Each country checked if the tracker information was correct for the areas where data were collected at the height of restrictions (Table 1)*.* Three categories of restrictions (low, moderate and high) were developed based on the variables deemed most influential on young children’s movement behaviours in this context. These were *ECEC closure* and *ability to go outside*. A low level of restriction was defined as pre-school being open or available to children of essential workers, and the ability to go out in public for exercise. A moderate level of restriction as ECEC being closed and the recommendation to limit time outside, for example only within immediate residential area, specific times of day, maintaining physical distancing etc. A high level of restriction was applied to countries where ECECs were closed and people were not allowed to go out in public to exercise.

**Table 1:** Key variables from the Oxford COVID-19 Government Response Tracker at the height of restrictions (OxCGRT)

Country	Preschool, nursery, school closures	Workplace closures	Public transport closure	Restrictions on domestic travel	Stay at home requirements	Limit on private gatherings (how many people can get together)	Ability to go outside for exercise*	Playground, skate park closures**
Australia	Kept open for children of essential workers.	Required closing (or work from home) for some sectors or categories of workers	No measures	Internal movement restrictions in place	Require not leaving house with exceptions for daily exercise, grocery shopping, and ‘essential’ trips	Restrictions on gatherings of 10 people or less	Yes - for exercise	All closed
Bangladesh	Required closing	Required closing (or work from home) for all-but-essential workplaces (eg grocery stores, doctors)	Required closing (or prohibit most citizens from using it)	Internal movement restrictions in place	Require not leaving house with exceptions for daily exercise, grocery shopping, and ‘essential’ trips	Restrictions on gatherings of 10 people or less	Not allowed	All closed
China	Required closing	Required closing (or work from home) for all-but-essential workplaces (eg grocery stores, doctors)	Required closing (or prohibit most citizens from using it)	Internal movement restrictions in place	Require not leaving house with minimal exceptions (eg allowed to leave once a week, or only one person can leave at a time, etc)	Restrictions on gatherings of 10 people or less	Yes - but encouraged to reduce if possible	Partial playground and skate park closures
Hong Kong	Required closing	Required closing (or work from home) for some sectors or categories of workers	No measures	No measures	Recommend not leaving house	There was no limit on how many people can congregate in private settings or at work, but not allowed to assemble in groups of more than 4 for public gatherings	Yes, but leisure venues for public gatherings were closed	All closed
India	Required closing	Required closing (or work from home) for all-but-essential workplaces (eg grocery stores, doctors)	Required closing (only open for emergency services)	Internal movement not allowed except for emergency with prior permission of police	Require not leaving house with minimal exceptions (eg allowed to leave once a week, or only one person can leave at a time)	Restrictions on gatherings of 10 people or less	Not Allowed	All closed
Indonesia	Required closing	Required closing (or work from home) for some sectors or categories of workers	Recommend closing (or significantly reduce volume /route/means of transport available)	Internal movement restrictions in place	Require not leaving house with exceptions for daily exercise, grocery shopping, and ‘essential’ trips	Restrictions on gatherings of 10 people or less	Yes - for exercise	All closed
Malaysia	Required closing	Required closing (or work from home) for all-but-essential workplaces (eg grocery stores, doctors)	No measures	Internal movement restrictions in place	Recommend not leaving house	Restrictions on gatherings between 50 people	Not allowed	All closed
Morocco	Stop teaching at all levels, public	Required closing (or work from home) for all-but-essential workplaces (eg grocery stores, doctors)	Required closing (or prohibit most citizens from using it)	limitation of travel between neighbourhood, regions and between cities	Leaving house not allowed with exceptions for health emergency, grocery shopping, and ‘essential’ trips	Prohibition of professional and private gatherings Professional meetings to be limited except nb. Limited and respecting the safety instructions	Not allowed	All closed
Pakistan	Required closing	Required closing (or work from home) for all-but-essential workplaces (eg grocery stores, doctors)	Required closing (or prohibit most citizens from using it)	Internal movement restrictions in place	Require not leaving house with exceptions for daily exercise, grocery shopping, and ‘essential’ trips	Restrictions on gatherings of 10 people or less	Allowed only until 5 pm	All closed
Spain	Required closing	Required closing (or work from home) for all-but-essential workplaces (eg grocery stores, doctors)	Recommend closing (or significantly reduce volume /route/means of transport available)	Internal movement restrictions in place	Require not leaving house with exceptions for grocery shopping and ‘essential’ trips	Restrictions on gatherings of 10 people or less	Not allowed	All closed
Sri Lanka	Required closing	Required closing (or work from home) for all-but-essential workplaces (eg grocery stores, doctors)	Required closing (or prohibit most citizens from using it)	Internal movement restrictions in place	Require not leaving house with minimal exceptions (eg allowed to leave once a week, or only one person can leave at a time)	Restrictions on gatherings of 10 people or less	Not allowed	All closed
Sweden	Preschool open.	Recommend closing (or recommend work from home)	No closure, however, fewer people per vehicle encouraged	Recommend not to travel between regions/cities (> 2 h from home)	No recommendation	Restrictions on gatherings between 50 people	Yes-allowed	All open
United States	Kept open for children of essential workers. The majority of children stayed at home	Required closing (or work from home) for all-but-essential workplaces (eg grocery stores, doctors)	Recommend closing (or significantly reduce volume /route/means of transport available)	Internal movement restrictions in place	Require not leaving house with exceptions for daily exercise, grocery shopping, and ‘essential’ trips	Restrictions on gatherings of 10 people or less	Yes - “Take a walk, ride your bike, hike, jog and be in nature for exercise — just keep at least 6 feet between you and others.”	All closed
Vietnam	Required closures	Required closing (or work from home) for all-but-essential workplaces (eg grocery stores, doctors)	Required closing (or prohibit most citizens from using it)	Internal movement restrictions in place	Require not leaving house with exceptions for daily exercise, grocery shopping, and ‘essential’ trips	Restrictions on gatherings of 2 people or less at public places outside workplace, schools, hospitals; 2 m distancing	Yes - but encouraged to reduce if possible	All closed

*As reported by the CI in each country. ** Based on a combination of ECEC closure and ability to go outside

### Measurement

Data collectors received training in how to administer the parent survey and use the REDCap [10] hosted at the University of Wollongong, Australia. Prior to participation, informed consent was obtained from the parents/legal guardians of participating children.

### Bias

The results presented are based on the report of the child’s primary carer, and as such there is a risk of recall bias. The internal validity of the dataset is strengthened given that for 86% of the sample, the same primary parent reported data at both time points. The response rate of 76% at T2 further reduces sample bias.

### Study size

Power calculations were performed for meeting TPA and SST guidelines. Due to the sampling methods we accounted for within person and within ECEC service dependence of the outcomes. The intra-class correlations within ECEC services and within children were estimated [14, 15] and then converted to random effect variances. For each generated data set, a multi-level model was fitted with random intercepts for ECEC service, children, and the additional random slope *β*_*k*_ for the change in outcomes from T1 to T2 in country k assuming that country effects varied according to the assumption ${\beta}_k\sim N\left(\beta, {\sigma}_{\beta}^2\right)$, with *β* the mean change effect across countries. Finally, the significance of parameter *β* was tested. The estimated power was ≥80% for meeting both the TPA and SST guidelines. All analyses were conducted using R [16] and R package lmer [17] with optimizer “bobyqa” used to fit the multi-level models. Missing data were ignored and not imputed; that is, missingness was assumed to be at random.

### Statistical methods

Descriptive data were calculated as frequencies (%) or means with 95% confidence intervals (CI, using Wald type confidence interval). Results were presented for the effect *β* as average or effect across countries with corresponding 95% CIs or as average mean change across countries with corresponding 95% CIs. Coefficients were based on a multi-level model with ECEC services as random effects (accounting for cluster sampling), children as random effects (accounting for paired data) and country random effects of change from T1 to T2.

A linear mixed model was fitted to the difference of outcomes at T2 relative to T1. Models similar to the multi-level models for the descriptive results were fitted without the child random effects, as there was only one observation per child. For the outcomes of meeting the guidelines at T2, a logistic multi-level regression model was fitted accounting for T1 with ECEC service and country sites as random effects.

## Results

### Participants

The final analytical sample comprised 948 respondents (children’s mean age T1 = 4.4 years, standard deviation (SD) = 0.6; T2 = 5.2 years, SD = 0.6). The average response rate for the sample was 76%, and the average time interval between T1 and T2 was 9.6 months (SD = 3.8). Of the children, 49% were female, 39% lived in rural areas, 71% were from LMICs, and 63% of parents had some level of tertiary education (Table S3). Eighty-three percent of children went outside during COVID-19 restrictions (see Table 2 for duration), and 59% lived in housing with access to an outside play area in the form of private garden/yard, or communal park/green or open space near their house (Table S4).

**Table 2:** Changes in young children’s movement behaviours and sleep characteristics before (T1) and during COVID-19 (T2)

	N	T1	T2	Mean change (95% CI)^**†**^			
				Unadjusted	***p***-value	Adjusted^§^	***p***-value
**Time spent in movement behaviours (min/day)**							
TPA	852	200.7 (5.0)	217.8 (4.8)	16.5 (−40.0,72.9)	0.540	25.1 (−31.7,81.9)	0.361
MVPA	847	60.6 (2.3)	55.6 (2.4)	−5.7 (−25.0,13.6)	0.528	5.6 (− 25.3,14.1)	0.552
SST	942	105.3 (3.6)	162.0 (4.2)	57.0 (43.0,71.0)	**< 0.0001**	54.9 (38.6,71.2)	**< 0.0001**
Total sleep duration - including nap (min)	946	664.7 (2.9)	641.2 (3.2)	−22.8 (−42.4,-3.2)	**0.026**	−9.2 (−28.9,10.6)	0.341
Nap duration (min)	287	111.5 (2.0)	97.4 (2.9)	−16.6 (−31.6,-1.6)	**0.034**	− 18.5 (− 33.6,-3.4)	**0.020**
**Proportion of children meeting the recommendations of WHO Global guidelines (%)**							
TPA	852	53.1 (2.0)	60.1 (1.7)	1.45 (0.56,3.72)	0.441	1.48 (0.56,3.87)	0.426
MVPA	847	50.8 (1.8)	48.7 (1.9)	0.82 (0.41,1.65)	0.577	0.83 (0.39,1.76)	0.633
SST	942	48.0 (1.8)	24.9 (1.7)	0.28 (0.17,0.46)	**< 0.0001**	0.31 (0.18,0.55)	**< 0.0001**
Sleep	946	84.2 (1.5)	79.3 (1.6)	0.67 (0.44,1.01)	0.055	0.89 (0.57,1.41)	0.628
All four recommendations	842	13.6 (1.4)	10.5 (1.2)	0.77 (0.40,1.48)	0.430	0.91 (0.44,1.88)	0.791
**Sleep characteristics**							
Bedtime (24Hr:Min)	947	21:20 (0:02)	22:01 (0:03)	0:40 (0:21,1:00)	**< 0.001**	0:34 (0:14,0:54)	**0.003**
Wake-time (24Hr:Min)	946	7:09 (0:02)	8:09 (0:03)	1:00 (0:37,1:23)	**< 0.0005**	0:59 (0:34,1:23)	**< 0.0005**
Poor sleep quality (%)	924	5.4 (1.0)	6.0 (0.9)	0.50 (0.19,1.29)	0.152	0.57 (0.21,1.54)	0.267
Use screen devices 2 h before bed (%)	922	72.5 (1.7)	65.0 (2.0)	0.66 (0.37,1.18)	0.165	0.76 (0.42,1.41)	0.388
**Outdoor time**							
Time spent outdoors on weekdays (min/day)	934	180.7 (4.6)	105.7 (3.7)	−75.7 (−141.6,-9.8)	**0.028**	−80.9 (−147.6,-14.1)	**0.021**
Time spent outdoors on weekends (min/day)	941	213.4 (5.0)	115.6 (4.0)	− 98.3 (− 160.3,-36.4)	**0.004**	−104.7 (− 166.7,-42.6)	**0.003**

*TPA* total physical activity, *MVPA* moderate-to-vigorous intensity physical activity, *SST* sedentary screen time

Data are presented as mean (standard error) for continuous variables or percentage (standard error) for categorical variables. Bold value indicates statistically significant effect (*p* < 0.05)

^†^Mean change effects are presented in min/day format for continuous variables or odds ratio for categorical variables.

^§^Adjusted for age by sex interaction, rurality, change in parent relationship to child (person who completed the survey), childcare centre (as random effects), country sites (as random effects), and parents’ highest level of education.

At the height of COVID-19 restrictions, 41% of the participants faced high, 46% moderate, and 13% low levels of restrictions. Fifty-three percent, 59, and 47% of parents were concerned about their child’s level of physical activity, sedentary behaviour and sleep, respectively. Around 80% of parents felt able to support their child to have healthy movement behaviours and had received resources from their child’s ECEC service and 62% had accessed online resources to support their child’s movement behaviours at home. Around one-third of parents reported that they felt more stressed and exhausted than before COVID-19 (Table S4).

Table 2 presents the changes in children’s movement behaviour patterns before and during COVID-19. Overall, children spent 55 min/day more (*p* < 0.0001) in SST and the proportion who met the SST guideline dropped from 48 to 25% (*p* < 0.0001). Children went to bed 34 min later (*p* = 0.003) and woke up 60 min later (*p* < 0.0005) than before COVID-19. The mean nap time decreased by 19 min/day (*p* = 0.020). Children spent 81 min (*p* = 0.021) and 105 min (*p* = 0.003) less time outdoors on weekdays and weekend days, respectively. No significant changes were observed in other behavioural variables.

Table 3 reports the associations between selected COVID-19 factors and changes in time spent in movement behaviours. Children whose parents were concerned about their child’s movement behaviours had a significantly greater increase in SST compared with parents who were not concerned (regression coefficient = 14.9; 95% CI 0.2–29.6). Children who lived in houses with outdoor spaces had a significantly greater increase in TPA (coefficient = 54.7; 95% CI 19.0–90.3)) and a smaller decrease in MVPA (−coefficient = 16.7; 95% CI 1.0–32.3)), compared with those with no outdoor space. Children whose parents reported receiving support from their ECEC service during COVID had a significantly smaller decrease in sleep duration (coefficient = 29.8; 95% CI 8.1–51.6) than those children whose service did not provide support to parents.

**Table 3:** Linear regression of associations between changes in movement behaviours and selected COVID-19 factors

COVID-19 factors	Changes in time spent in movement behaviours							
	TPA		MVPA		SST		Sleep duration	
	Model 1	Model 2	Model 1	Model 2	Model 1	Model 2	Model 1	Model 2
Level of restrictions								
High (Ref)	–	–	–	–	–	–	–	–
Low	6.3 (− 185.1197.7)	52.9 (− 160.0,265.8)	27.7 (−25.2,80.7)	36.9 (−31.4105.2)	−9.7 (−50.0,30.6)	−4.6 (−60.3,51.1)	− 14.6 (− 70.1,40.9)	−10.4 (− 98.1,77.4)
Moderate	− 17.7 (− 193.1157.7)	10.1 (− 137.0,157.2)	4.4 (− 43.9,52.7)	6.3 (− 40.8,53.3)	−18.0 (− 52.8,16.7)	− 22.5 (− 59.2,14.2)	−4.7 (− 52.4,43.0)	− 28.2 (− 83.6,27.2)
Country income level								
High-income (HIC) (Ref)	–	–	–	–	–	–	–	–
Low/middle-income (LMIC)	44.1 (− 96.0,184.2)	78.1 (−96.7252.9)	0.7 (− 41.9,43.4)	22.6 (−33.2,78.5)	−11.5 (− 41.9,18.9)	− 10.9 (− 54.4,32.6)	21.5 (− 17.7,60.6)	21.8 (− 48.7,92.3)
Go outside during COVID-19								
No (Ref)	–	–	–	–	–	–	–	–
Yes	7.1 (−22.6,36.9)	9.3 (−20.9,39.4)	5.5 (− 7.2,18.3)	6.0 (− 7.2,19.2)	2.0 (− 16.1,20.1)	5.8 (− 12.9,24.5)	4.1 (− 11.6,19.7)	4.1 (− 11.6,19.7)
Parent’s concern about child’s movement behaviour^§^								
No (Ref)	–	–	N/A	N/A	**–**	**–**	–	–
Yes	−24.1 (− 48.2,0.06)	− 23.0 (− 47.4,1.4)			**15.7 (1.6,19.7)**	**14.9 (0.2,29.6)**	4.7 (−8.2,17.7)	4.3 (− 8.9,17.5)
Parent’s perceived ability to support child to have healthy movement behaviours								
No (Ref)	–	–	–	–	–	–	–	–
Yes	−7.8 (−36.6,21.0)	−10.2 (− 39.0,18.5)	8.6 (− 3.9,21.0)	7.4 (−5.2,20.0)	−10.1 (− 27.1,6.9)	− 4.4 (− 21.8,13.1)	5.6 (− 9.2,20.4)	5.7 (− 9.1,20.4)
Presence of outdoor space within house compound								
No (Ref)	**–**	**–**	–	–	–	–	–	–
Yes	**50.9 (16.5,85.4)**	**54.7 (19.0,90.3)**	**17.1 (2.5,31.8)**	**16.7 (1.0,32.3)**	−15.6 (− 33.9,2.8)	−16.9 (−37.6,3.8)	1.6 (− 15.6,18.7)	1.0 (− 17.0,19.0)
Number of adults living within the same household								
Two or less (Ref)	–	–	–	–	–	–	–	–
More than two	5.3 (−18.1,28.6)	3.9 (−19.4,27.2)	−2.6 (−12.8,7.6)	−3.3 (−13.7,7.1)	1.5 (−15.3,12.3)	−0.8 (− 15.2,13.6)	−6.3 (− 18.4,5.7)	−9.4 (− 21.7,2.9)
Number of children living within the same household								
Two or less (Ref)	–	–	–	–	–	–	–	–
More than two	22.5 (−6.6,51.6)	25.2 (−4.1,54.4)	2.5 (−10.1,15.1)	3.2 (−9.7,16.1)	−2.5 (− 19.3,14.2)	0.0 (−17.3,17.3)	−2.6 (− 17.2,12.0)	−2.6 (− 17.3,12.1)
Parent’s perceived level of stress compared to before COVID-19								
Less/about the same (Ref)	–	–	–	–	–	–	–	–
More stressed	−11.5 (−36.4,13.5)	−1.4 (−29.6,26.8)	−4.9 (− 15.7,5.9)	− 8.4 (− 21.0,4.1)	**15.0 (0.4,29.6)**	8.2 (− 8.8,25.3)	−1.7 (− 14.5,11.1)	4.0 (− 10.5,18.5)
Parent’s perceived level of exhaustion compared to before COVID-19								
Less/about the same (Ref)	–	–	–	–	–	–	–	–
More exhausted	− 10.6 (−35.2,14.0)	−5.4 (− 33.2,22.3)	3.8 (− 6.8,14.4)	8.2 (− 4.0,20.4)	13.3 (−1.2,27.8)	6.1 (− 10.5,22.7)	**−12.8 (− 25.4,-0.2)**	−13.3 (− 27.4,0.7)
Receiving any support from childcare centre								
No (Ref)	–	–	–	–	–	–	**–**	**–**
Yes	− 8.2 (− 51.5,35.1)	− 8.2 (− 53.7,37.4)	− 2.0 (− 20.0,16.0)	−0.1 (− 19.4,19.1)	− 22.6 (− 45.9,0.7)	− 21.7 (− 47.5,4.1)	**22.3 (1.2,43.4)**	**29.8 (8.1,51.6)**
Using any resources to support/facilitate child’s movement behaviours at home								
No (Ref)	–	–	–	–	–	–	–	–
Yes	2.7 (−23.2,28.5)	7.2 (− 19.0,33.5)	0.8 (− 10.3,11.9)	0.3 (− 11.2,11.8)	4.4 (− 11.0,19.9)	3.2 (− 13.0,19.5)	4.8 (− 8.9,18.6)	5.0 (− 8.9,18.9)

*TPA* total physical activity, *MVPA* moderate-to-vigorous intensity physical activity, *SST* sedentary screen time

Data are presented as unstandardized regression coefficients (95% confidence interval); bold value indicates statistically significant effect

Model 1: Adjusted for age (at T2) by sex interaction, change in age (T2 relative to T1), rurality, change in parent relationship to child (person who completed the survey), childcare centre (as random effects), country sites (as random effects), parents’ highest level of education, and children who reported as sick at T2 (preventing from being active at T2)

Model 2: Included all variables in Model 1 and all COVID-19 factors

^§^Separate questions were asked for total physical activity, sitting (including screen time) and sleep, but not for MVPA

Table 4 reports the associations between selected COVID-19 factors and meeting the WHO Global guidelines during COVID-19. Only the factors that showed a significant association in the fully adjusted models (Table 4, Model 2) are reported. Compared with countries with a high level of restrictions, children in countries with a low level were 3.59 times more likely to meet the MVPA guideline (95%Confidence Interval [CI] 1.39, 9.30) and 6.71 times more likely to meet the sleep guideline (95%CI 1.77, 25.46); whereas children in countries with a moderate level of restrictions were 2.71 times more likely to meet the SST guideline (95%CI 1.56, 4.70). Children from LMICs were 12.17 times more likely to meet the TPA (95%CI 3.03, 49.00), 1.96 times more likely to meet the MVPA (95%CI 1.02, 3.77) and 2.16 times more likely to meet the SST guidelines (95%CI 1.19, 3.94) than children from HICs. Compared with children who were not allowed to go outside, those who were allowed were 3.3 times more likely to meet all four guidelines (95%CI 1.12, 9.76) and 1.7 times more likely to meet the TPA guideline (95%CI 1.05, 2.75).

**Table 4:** Logistic regression of associations between meeting WHO Global guidelines and selected COVID-19 factors

	TPA guideline		MVPA guideline		SST guideline		Sleep guideline		All guidelines	
	Model 1	Model 2	Model 1	Model 2	Model 1	Model 2	Model 1	Model 2	Model 1	Model 2
Level of restrictions										
High (Ref)	1.00	1.00	1.00	1.00	1.00	1.00	1.00	1.00	1.00	1.00
Low	0.94 (0.11,7.83)	4.29 (0.74,24.90)	**4.43 (1.77,11.12)**	**3.59 (1.39,9.30)**	1.13 (0.52,2.44)	1.91 (0.79,4.60)	**9.50 (2.60,34.71)**	**6.71 (1.77,25.46)**	2.11 (0.98,4.54)	4.33 (0.92,20.39)
Moderate	0.44 (0.06,3.01)	0.50 (0.15,1.65)	0.67 (0.30,1.47)	0.89 (0.49,1.62)	**1.98 (1.08,3.62)**	**2.71 (1.56,4.70)**	1.18 (0.48,2.89)	0.76 (0.38,1.54)	0.99 (0.47,2.07)	1.14 (0.47,2.78)
Country income level										
High-income (HIC) (Ref)	1.00	1.00	1.00	1.00	1.00	1.00	1.00	1.00	1.00	1.00
Low/middle-income (LMIC)	3.87 (0.81,18.53)	**12.17 (3.03,49.00)**	0.67 (0.23,1.94)	**1.96 (1.02,3.77)**	**1.98 (1.09,3.60)**	**2.16 (1.19,3.94)**	0.44 (0.15,1.31)	1.21 (0.55,2.65)	1.30 (0.57,2.97)	3.17 (0.86,11.75)
Go outside during COVID-19										
No (Ref)	1.00	1.00	1.00	1.00	1.00	1.00	1.00	1.00	1.00	1.00
Yes	**1.75 (1.10,2.78)**	**1.70 (1.05,2.75)**	**1.70 (1.06,2.71)**	1.52 (0.95,2.45)	1.20 (0.76,1.89)	1.19 (0.75,1.89)	1.53 (0.93,2.51)	1.51 (0.92,2.45)	**4.21 (1.46,12.18)**	**3.30 (1.12,9.76)**
Parent’s concern about child’s movement behaviour^§^										
No (Ref)	1.00	1.00	N/A	N/A	1.00	1.00	1.00	1.00	N/A	N/A
Yes	**0.49 (0.34,0.72)**	**0.50 (0.34,0.74)**			**0.60 (0.42,0.86)**	**0.68 (0.47,0.96)**	1.00 (0.65,1.56)	0.97 (0.63,1.50)		
Parent’s perceived ability to support child to have healthy movement behaviours										
No (Ref)	1.00	1.00	1.00	1.00	1.00	1.00	1.00	1.00	1.00	1.00
Yes	1.25 (0.77,2.01)	1.15 (0.71,1.86)	**2.19 (1.35,3.55)**	**1.89 (1.17,3.06)**	1.15 (0.74,1.79)	1.14 (0.73,1.79)	0.99 (0.61,1.59)	0.87 (0.54,1.41)	1.56 (0.71,3.45)	1.14 (0.51,2.51)
Presence of outdoor space within house compound										
No (Ref)	1.00	1.00	1.00	1.00	1.00	1.00	1.00	1.00	1.00	1.00
Yes	**1.88 (1.07,3.30)**	**2.41 (1.32,4.41)**	**2.61 (1.55,4.40)**	**2.61 (1.56,4.36)**	1.29 (0.81,2.06)	1.14 (0.70,1.87)	0.80 (0.46,1.38)	0.65 (0.38,1.11)	**2.83 (1.43,5.60)**	1.82 (0.78,4.23)
Number of adults living within the same household										
Two or less (Ref)	1.00	1.00	1.00	1.00	1.00	1.00	1.00	1.00	1.00	1.00
More than two	**1.64 (1.12,2.42)**	**1.55 (1.04,2.33)**	0.98 (0.68,1.39)	0.97 (0.67,1.40)	0.98 (0.70,1.37)	0.96 (0.68,1.36)	1.04 (0.70,1.53)	1.12 (0.76,1.66)	0.96 (0.54,1.70)	1.06 (0.58,1.94)
Number of children living within the same household										
Two or less (Ref)	1.00	1.00	1.00	1.00	1.00	1.00	1.00	1.00	1.00	1.00
More than two	1.21 (0.74,1.98)	1.27 (0.76,2.13)	1.01 (0.66,1.56)	0.92 (0.59,1.45)	0.99 (0.65,1.50)	0.85 (0.56,1.31)	0.99 (0.62,1.58)	1.01 (0.63,1.61)	1.42 (0.76,2.63)	1.22 (0.64,2.33)
Parent’s perceived level of stress compared to before COVID-19										
Less/about the same (Ref)	1.00	1.00	1.00	1.00	1.00	1.00	1.00	1.00	1.00	1.00
More stressed	1.03 (0.69,1.53)	1.41 (0.87,2.28)	1.04 (0.71,1.52)	0.98 (0.63,1.54)	**0.65 (0.45,0.93)**	0.90 (0.59,1.37)	0.86 (0.56,1.33)	1.06 (0.65,1.73)	**0.50 (0.26,0.93)**	0.56 (0.26,1.18)
Parent’s perceived level of exhaustion compared to before COVID-19										
Less/about the same (Ref)	1.00	1.00	1.00	1.00	1.00	1.00	1.00	1.00	1.00	1.00
More exhausted	0.73 (0.50,1.06)	0.65 (0.41,1.02)	1.18 (0.82,1.70)	1.17 (0.75,1.83)	**0.63 (0.43,0.90)**	0.74 (0.49,1.13)	**0.62 (0.41,0.94)**	**0.59 (0.37,0.95)**	0.71 (0.39,1.30)	1.16 (0.57,2.33)
Receiving any supports from childcare centre										
No (Ref)	1.00	1.00	1.00	1.00	1.00	1.00	1.00	1.00	1.00	1.00
Yes	1.23 (0.63,2.39)	1.33 (0.67,2.64)	1.55 (0.85,2.85)	1.64 (0.88,3.09)	1.16 (0.66,2.05)	1.18 (0.64,2.16)	1.82 (0.96,3.46)	1.64 (0.87,3.09)	1.76 (0.66,4.70)	1.47 (0.51,4.25)
Using any resources to support/facilitate child’s movement behaviours at home										
No (Ref)	1.00	1.00	1.00	1.00	1.00	1.00	1.00	1.00	1.00	1.00
Yes	1.12 (0.77,1.64)	1.16 (0.78,1.72)	1.39 (0.93,2.08)	1.40 (0.93,2.12)	0.89 (0.61,1.28)	0.99 (0.68,1.43)	1.11 (0.67,1.82)	1.05 (0.65,1.71)	0.98 (0.54,1.77)	1.15 (0.63,2.09)

*TPA* total physical activity, *MVPA* moderate-to-vigorous intensity physical activity, *SST* sedentary screen time

Data are presented as odds ratios (95% confidence interval); bold value indicates statistically significant effect

Model 1: Adjusted for age (at T2) by sex interaction, rurality, childcare centre (as random effects), country sites (as random effects), parents’ highest level of education, children who reported as sick at T2 (preventing from being active at T2), and corresponding T1 measures (e.g., meeting/not meeting TPA guideline at T1 for TPA model)

Model 2: Similar to Model 1 but also included other COVID-19 factors

^§^Separate questions were asked for total physical activity, sitting (including screen time) and sleep, but not for MVPA

Compared with parents who were not concerned about their child’s movement behaviours, children of parents who were concerned were half as likely to meet the TPA (95%CI 0.34, 0.74) and two-thirds less likely to meet the SST guidelines (95%CI 0.47, 0.96). However, children whose parents believed they had the ability to support their child’s movement behaviours were 1.89 times more likely to meet the MVPA guidelines compared with those who did not believe this (95%CI 1.17, 3.06). Compared with children who lived in houses with no outdoor play spaces, children in houses with outdoor play spaces were 2.41 times more likely to meet the TPA (95%CI 1.32, 4.41) and 2.61 times more likely to meet the MVPA guidelines (95%CI 1.56, 4.36). Children whose parents felt more exhausted at the time of the survey compared with before COVID-19 were 0.59 times less likely to meet the sleep guideline than children whose parents felt less or the same level of exhaustion (95%CI 0.37, 0.95).

## Discussion

The purpose of this study was to examine how, compared with the time period pre-COVID, the restrictions of the COVID-19 pandemic influenced physical activity, sedentary screen time and sleep among pre-schoolers.

### Key results summary

We hypothesised that, compared with pre-COVID-19, there would be increases in SST, decreases in PA and changes in sleep patterns among young children at the height of COVID-19 restrictions. These hypotheses were confirmed except for PA levels. Family and community factors that were associated with changes in movement behaviours included the presence of an outdoor space to play within the home compound, a supportive ECEC service, and parental stress and exhaustion levels. Additional factors that were associated with a greater likelihood of meeting WHO Global guidelines during COVID-19 included living in a LMIC, living in a country with lower levels of restrictions, being able to go outside, having more than two adults living in the house, and having parents who were less concerned about their child’s movement behaviours.

The small changes in PA were surprising and contrary to findings that have reported large declines in PA during the pandemic period among older children [12, 18, 19]. These differences may be explained methodologically. Our study used a longitudinal design with parents reporting their children’s current activity levels at a particular point in time while the others were cross-sectional where parents retrospectively reported what changes may have occurred from pre-COVID to during COVID-19, often in the form of Likert scale (more or less active than before), without parents having to quantify the changes. Data reported this way has a greater chance of emotional bias (parents more likely to see things negatively compared with before), especially given the higher level of parent stress and exhaustion during COVID-19. These smaller changes can be explained by more parents being at home during COVID-19 and spending more time with their child, and children getting more time to play due to closure of ECEC services. As children in our study were not attending ECEC and not participating in any structured learning activities they had more time for active play and in home environments that were more conducive to promoting activity than the environments in ECEC services.

The significant increase in SST during the COVID-19 period is consistent with other studies among children [12, 18–20]. This is largely due to children spending less time outdoors and in some countries undertaking activities online while at home. Other factors might include parents working from home and using electronic media devices to keep their child busy while they worked. A concern here is that only half the children met the SST guidelines before COVID-19. This proportion almost halved during the pandemic, thereby considerably increasing sedentary time in addition to the increase in screen-time. Although not all SST is detrimental [21] (e.g educational apps, face-time with relatives), the low compliance with SST recommendations during COVID should raise concern for the potential negative consequences for young children that may result from engaging in SST in excess of the guidelines, both from a screen time and sedentary time perspective.

There was no change in total sleep duration, unlike among school-aged children and youth who reportedly slept more during the restriction [12]. Reasons for the observed changes in sleep patterns may include children choosing not to nap at home, unlike when attending ECEC where they are required to nap or rest quietly. Parents reported that children slept for longer in the morning, were less tired in the afternoon and therefore they did not nap. There was probably more flexibility around bedtime, with parents allowing their child to go to bed slightly later. These patterns are consistent with what is observed during holiday periods and on weekends [22].

Children spent less time outdoors on weekdays and weekend days during the pandemic. This is consistent with other studies of children and youth during the pandemic [12, 18]. Time spent outdoors provides many benefits for children and their parents including higher levels of PA [23]; indeed, in those countries where children were able to play outdoors, higher levels of PA were reported. Policies that allow children to play outdoors while at the same time minimising the risk of transmission of infection are needed. Wearing masks and enforcing physical distancing in playgrounds, parks and other green spaces may provide a solution to this challenge.

Our results highlight the important role parents play in supporting their child to participate in healthy levels of movement behaviours. Parents of children who were less likely to meet SST or PA guidelines, were more concerned about their children’s movement behaviours than parents whose children met the guidelines. This highlights that even though parents were aware that these behaviours were being compromised because of COVID-19, and were concerned by this, they were not necessarily in a position to address their concerns. This may be due to work/time constraints or parental stress and exhaustion. These parents’ concerns may have been further perpetuated if they were spending more time at home with their children than usual and so were acutely aware of their children’s movement behaviours.

However, children whose parents believed they had the ability to support their child were more likely to meet the MVPA guidelines compared with those who did not believe this. It is plausible that these parents were already actively encouraging their children to increase movement, thus reporting a significant amount of time spent in MVPA [12, 24]. This distinction between the influence of parents’ reported concern (their belief), versus their ability to support their child’s movement behaviours (their behaviour), raises questions regarding how best to support parents to encourage children’s movement behaviours during times of increased stress and exhaustion such as during COVID-19. This association between parent concern and children’s movement behaviours is encouraging because it shows that parents are aware of the importance of healthy levels of these behaviours at this age and of the need to support their child in meeting guidelines.

One-third of parents reported feeling more stressed and exhausted during COVID-19, compared with the period prior to the pandemic. High levels of parental stress and exhaustion were related to poorer movement behaviour outcomes. This is not surprising with ECEC services closed in many countries and parents juggling working from home and educating their children during this period. An association between the struggle to handle childcare responsibilities during COVID-19 and heightened levels of stress and exhaustion within the home environment [25]. Combined with not being able to go outside as normal or children not able to play with their friends creates a “perfect storm” for higher levels of parental stress and exhaustion. It is possible that parent exhaustion, and children’s change in sleep, could be attributed to any number of heightened stressors during the pandemic, with family downtime and typical routines thrown out of balance. For instance, engagement with social media and other communication technologies during the pandemic may result in undue stress and anxiety for both parents and children, with potential lasting impacts on daily routines [20].

Children in LMICs were more likely to meet movement behaviour guidelines then those in HICs. All countries with high levels of restrictions were LMICs. Higher levels of restriction were associated with lower movement behaviours, but living in an LMIC was associated with a higher likelihood of meeting PA and SST guidelines. This was due to children in some LMICs leaving the cities to spend time with relatives in rural areas, particularly during school holidays, where they had more access to outdoor spaces and enforcement of COVID restrictions were not as strict. With less restriction on their ability to go outside, these children were likely to be adequately active and less engaged in screen time.

Children living in countries with low or moderate restrictions were more likely to meet the guidelines for all three movement behaviours, compared with children living in countries with high levels of restrictions. These finding suggest that there is an interrelationships among the movement behaviours and that COVID restrictions can have an impact on children across the entire day. Not being able to go outside greatly reduces the opportunities children have for PA, and consequently children’s engagement in SST increases. Lower levels of PA and higher levels of SST in these children results in shorter total sleep durations.

### Generalisability

We found that when examining all the countries collectively there were no significant differences in the proportion of children who met all four movement behaviour guidelines during COVID compared to before the pandemic. Of the individual guidelines, only SST increased during the pandemic. As convenience sampling was used, these results are not generalisable beyond the study participants.

### Limitations

A limitation of the study was that movement behaviours were captured via parent report and not objectively measured. While the coefficients obtained from the concurrent validity of the PA questions against accelerometry were low, they are typical for what one might see for a PA question vs accelerometry [26]. Although 72-h accelerometry data for Time 1 was available for comparison, given the range of severity of the pandemic restrictions in participating countries, it was determined by the authors that it would not be feasible to collect accelerometer data across all settings at the height of the pandemic (T2). There is a possibility that parents may have under-reported children’s PA levels before COVID-19, as they may not have been aware of the extent of their child’s PA in the ECEC setting as well as due to limited time spent with their children.

At both time-points parent completed the survey via interview in the majority of cases (54.5% at T1 and 90.7% at T2). While in many countries, the same research teams collected data at T1 and T2 and no differences in participants’ willingness to answer the survey was reported during data collection, there may have been some degree of social desirability response bias in the parent reporting of their child’s movement behaviours before and during COVID. This may be particularly evident among parents who completed the survey via interview. However, since parents were not informed of the WHO Global guidelines prior to reporting their child’s movement behaviours, they may not have felt the need to report against ideal levels of movement behaviours, thereby reducing the likelihood of social desirability bias [27]. While the overall sample size was close to 1000, it was small in a number of countries and the children who participated are not necessarily representative of a broader population.

### Implications for future research, policy and practice

- The strength of the associations between levels of government restrictions and all three movement behaviours highlights that priority must be given to keeping ECEC services open if it is feasible to do so in a COVID-safe manner. This will support children to accrue the recommended movement behaviours.
- Being allowed outside during times of movement restrictions was independently associated with meeting all guidelines, emphasising the importance of i) allowing people to go outside each day, ii) parents providing opportunities for children to be outside, and iii) promoting active play for children when outside, with appropriate risk-mitigation strategies.
- The findings highlight the importance of access to public green spaces, particularly in densely populated urban areas and for those without a garden/yard attached to their home. This has implications for land use policy, urban planning and housing design and development (e.g. rooftop gardens).
- Having parents and children spending more time at home during times of movement restrictions, provides opportunities for parents to participate in physical activity together with their child. This could potentially result in improved mental health outcomes and increased PA for parents, coupled with increased PA and reduced SST for children [28, 29]. It would also encourage social interaction and enjoyment among children and their parents.
- Developing resources that provide suggestions for ways to incorporate both indoor and outdoor physical activity, reduce SST and facilitate heathy levels of sleep, during times of movement restrictions could be particularly beneficial for both parents and children
  - Outdoor activities could include a nature walk or time spent at the playground, in the park/field playing tag, ball games or imaginative free play.
  - Indoor activities that do not require a lot of equipment and can keep children entertained for a considerable time include building a playhouse using blankets, boxes or towels, soft balls, playing hide and seek, or dancing to music.
  - Adapting equipment for small indoor spaces could include using a balloon instead of a ball, or using a skipping rope instead of running, or household items such as rolled up socks and a basket.
  - Co-viewing and interacting with children during SST and selecting educational content and active engagement over passive viewing is recommended [21].
  - Try to maintain regular bed and wake times. Be mindful that going to bed later (and waking up later), while still resulting in the same sleep duration may not be as beneficial as going to bed earlier (and waking up earlier). Keeping children active during the day, will also ensure they are tired and able to keep to regular sleep times.
- Parental mental health needs to be considered when deciding what level of restrictions to impose to control the spread of COVID-19 and the consequences such restrictions may have on other aspects of health. In countries where levels of restrictions were low, parents’ stress and exhaustion were not as high, reaffirming that such restrictions impact parents, which may subsequently impact the child. Policymakers need to consider this in future pandemics or in subsequent waves of COVID-19 when making decisions about whether to close ECEC services or to adopt strict home quarantine orders.
- The aggregated data across the 14 countries suggested that there was not a considerable change in the children’s overall levels of PA (TPA and MVPA) from before to during the COVID-19 pandemic. Further research is required to explore differences in PA at the individual country level, to determine how the associated factors vary across different contexts and levels of restrictions and what the implications of this are.

## Conclusion

This study is unique as it reports on longitudinal changes in movement behaviour in a diverse, international population of young children from urban and rural locations, and the influence of movement restriction imposed by governments in response to the COVID-19 pandemic. Eight of the countries and 71% of participants are low- or middle-income; we know very little about movement behaviours or the impact of COVID-19 in these countries. This study presents a magnitude of changes in movement behaviours, adding to existing findings reporting parents’ perceptions of changes in movement behaviours during the pandemic.

With PA and SST levels of children from LMICs less impacted by COVID-19 than HICs, the results highlight that factors, which influence healthy levels of movement behaviours, differ between HICs and LMICs and consequently the implications vary. Policies and efforts therefore need to be specific to each country’s context. We suggest that future international studies present results that disaggregated by regions or income levels to best capture this variability.

Children in disadvantaged communities who do not have access to an outdoor space could be particularly affected as these areas become critically important during periods when restrictions are enforced. These findings can inform efforts to support parents of young children to promote a healthy balanced pattern of movement behaviours during the COVID-19 outbreak, the recovery phase and future pandemics. Parents’ stress and exhaustion levels and their self-efficacy to support healthy movement behaviour patterns in their children needs to be addressed.

## Supplementary Information

**Additional file 1: Table S1**: Parent/Caregiver Survey. **Table S2**. Descriptive characteristics of participating countries. **Table S3**. Characteristics of analytic sample. **Table S4**. Distribution of analytic sample according to COVID-19 factors

## Data Availability

The data that support the findings of this study are available from participating country contacts but restrictions apply to the availability of these data, which were used under license for the current study, and so are not publicly available. Data are however available from the authors upon reasonable request and with permission of all participating country contacts and the relevant ethics committees.
