# Supplementary material for: Global effect of COVID-19 pandemic on physical activity, sedentary behaviour and sleep among 3- to 5-year-old children: a longitudinal study of 14 countries
Source: BMC Public Health. 2021 May 17;21:940. doi: 10.1186/s12889-021-10852-3 (PMC8128084; doi:10.1186/s12889-021-10852-3)
Supplement: Supplementary file 1 — Additional file 1: Table S1: Parent/Caregiver Survey. Table S2. Descriptive characteristics of participating countries. Table S3. Characteristics of analytic sample. Table S4. Distribution of analytic sample according to COVID-19 factors [file 12889_2021_10852_MOESM1_ESM.docx]

**Global effect of COVID-19 pandemic on physical activity, sedentary behaviour and sleep among 3- to 5-year-old children: a longitudinal study of 14 countries**

**Authors:** Anthony D Okely, Katharina E Kariippanon, Hongyan Guan, Ellie K. Taylor, Thomas Suesse, Penny L Cross, Kar Hau Chong, Adang Suherman, Ali Turab, Amanda E. Staiano, Amy S Ha, Asmaa El Hamdouchi, Aqsa Baig, Bee Koon Poh, Borja Del Pozo Cruz, Cecilia H. S. Chan, Christine Delisle Nyström, Denise Koh, E. Kipling Webster, Himangi Lubree, Hong Kim Tang, Issad Baddou, Jesus del Pozo-Cruz, Jyh Eiin Wong, Kuston Sultoni, Maria Nacher, Marie Löf, Mingming Cui, Mohammad Sorowar Hossain, P. W. Prasad Chathurangana, Uddhavi Kand, V. Pujitha Wickramasinghe, Rebecca Calleia, Shameema Ferdous, Thanh Van Kim, Xiaojuan Wang, Catherine E Draper.

**Table S1: Parent/Caregiver Survey**


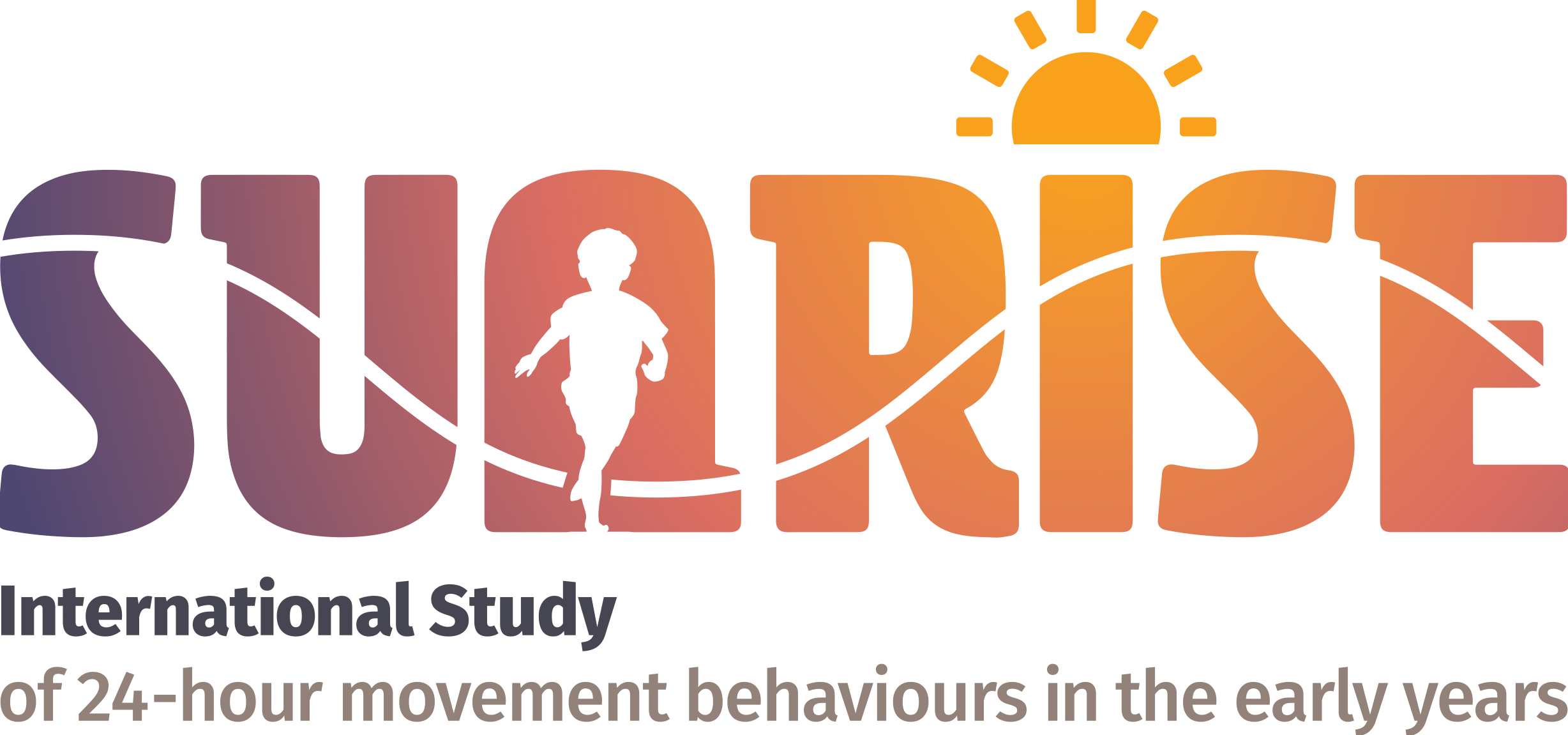


Office use only

Child ID: __ __ __ __ __ __ __

**THE SUNRISE STUDY**

**INTERNATIONAL STUDY OF 24-HOUR MOVEMENT BEHAVIOURS IN THE EARLY YEARS**

**PARENT/CAREGIVER SURVEY**

*This questionnaire is to be administered/completed by the MAIN caregiver of the child who lives with them and has previously completed this questionnaire. A separate questionnaire should be used for each eligible child who participated.*

This survey asks you about your child’s movement behaviours (physical activity, sedentary behaviour, screen time and sleep) **during COVID-19 related restrictions in your country.** If restrictions have been lifted or minimized, we ask you to consider your child’s movement behaviours for a typical week when **all** restrictions were in place.

| **CHILD AND CAREGIVER BACKGROUND** | | | | | | |
| --- | --- | --- | --- | --- | --- | --- |
| **1.** | | Parent/caregiver's relationship to the child participating in the study? | | | | |
|  | | Mother  Father  Grandmother  Grandfather  Legal Guardian  Other (please specify): ……………………………………………….. | | | | |
| **2.** | | Was your child sick in the last week, or did anything prevent [him / her] from being active at home in the last week?  🞏 Yes 🞏 No  **If yes, go to 2a** | | | | |
| **2a.** | | **If Yes,** what prevented your child from being active?  ___________________________________________ | | | | |
| **PHYSICAL ACTIVITY, SEDENTARY BEHAVIOUR, SCREEN TIME AND SLEEP** | | | | | |  |
| The next questions ask about your child's movement behaviours during COVID-19 related restrictions. Please report the **number of hours and minutes per day** (for all the questions). E.g. 1 hour and 30 mins | | | | | |  |
| **3.** | | In a typical 24 hour period during COVID-19 restrictions, how much time did the child who is participating in this study spend: (Please record these as accurately as you can to the nearest minute) | | | |  |
| **3a.** | | In a variety of physical activities, spread throughout the day?  For example: active play, running, playing with balls, moving to music/dancing, swimming, riding a scooter/tricycle/bike. | …..…..hrs ……....mins | | |  |
| **3b.** | | In energetic play that causes him/her to 'huff and puff' and increases his/her heart rate (this would be part of the total time spent in a variety of activities just mentioned). | …..…..hrs ……....mins | | |  |
| **3c.** | | Using any electronic screen device such as a smart phone, tablet, video game, or watching television or movies, videos on the internet while they were sitting or lying down? | …..…..hrs ……....mins | | |  |
| **4.** | | On a typical **weekday** during COVID-19 restrictions, how much time did this child spend outside? | …..…..hrs ……....mins | | |  |
| **5.** | | On a typical **weekend day** during COVID-19 restrictions, how much time did this child spend outside? | …..…..hrs ……....mins | | |  |
| **6.** | | In a typical week during COVID-19 restrictions, did your child use electronic screen devices (e.g. TV, video game, computer, tablet or smartphone) in the 2 hours before bedtime on a daily basis?  **If no, go to question 7** | | | |  |
|  | | Yes  No  Don’t know | | | |  |
| **6a.** | | **If Yes**, how close to bedtime did your child usually use these devices? | | | |  |
|  | | Closer than 30 minutes before bedtime  30 mins to less than 1 hour before bedtime  Between 1 and 2 hours before bedtime | | | |  |
| **7.** | | In a typical week during COVID-19 restrictions, did your child have electronic screen devices in the room where he/she sleeps (e.g. TV, video game, computer, tablet or smartphone)? | | | |  |
|  | | Yes  No | | | |  |
| **8.** | | How many hours of sleep did your child get in a typical 24-hours day (**including naps**) during COVID-19 restrictions? | …..…..hrs ……....mins | | |  |
| **9.** | | In a typical week during COVID-19 restrictions, did your child nap? | | | |  |
|  | | Yes  No  **If no, go to 10** | | | |  |
| **9a.** | | What time did your child nap? | | | |  |
|  | | Begin time: ……………………. End time: ……………………… | | | |  |
| **10.** | | In a typical week during COVID-19 restrictions, did the child have a consistent bedtime? | | | |  |
|  | | Yes, bedtime did not vary by more than 30 minutes each day  No, bedtime varied more than 30 minutes each day | | | |  |
| **11.** | | In a typical week during COVID-19 restrictions, did your child have a consistent wake-up time? | | | |  |
|  | | Yes, wake-up time did not vary by more than 30 minutes each day  No, wake-up time varied more than 30 minutes each day | | | |  |
| **12a.** | | In a typical week during COVID-19 restrictions, what time did your child go to bed at night? | | | |  |
|  | | Bed time: ……………………. PM | | | |  |
| **12b.** | | In a typical week during COVID-19 restrictions, what time did your child get up in the morning? | | | |  |
|  | | Wake-up time: ……………………… AM | | | |  |
| **13.** | | On a scale of 1 to 7, with the higher number indicating higher quality, how would you rate the quality of this child's sleep **in a typical week during COVID-19 restrictions**?  **1 would indicate** very difficult to settle, wakes many times during the night for prolonged periods and is very restless (tosses and turns, throw off bedclothes) **while 7 would indicate** settles and drifts off to sleep within a few minutes, sleeps right through the night, and has a very sound, deep sleep) | | | |  |
|  | | 1  2  3  4  5  6  7  Don’t know | | | |  |
| **COVID-19:** | | | | | |  |
| **14.** | | **Due to the impact of COVID-19**, how long has your child stayed at home without going to their childcare centre/pre-school/kindergarten? | | | | |
|  | | Less than one week  1 to 4 weeks  5 to 8 weeks  More than 8 weeks | | | | |
| **15.** | | | **If you are/were allowed to go outside during COVID-19 restrictions**, did your child play/go: **(tick as many as appropriate)**  On the property (ie. Garden/yard)  To a friend/relative’s home  To a park/square or playground  To a swimming pool/creek/river/dam/waterhole  To the street  To walk a pet  Any other not mentioned? (please specify)  …………………………………………………………………………………………………….…………………………………………  None | | |  |
| **16.** | | | **During COVID-19 restrictions**, have you been concerned about the level of physical activity, sitting (including screen time) or sleep your child participates in? | | |  |
|  | | | Physical activity 🞏 Yes 🞏 No  Sitting (including screen time) 🞏 Yes 🞏 No  Sleep 🞏 Yes 🞏 No | | |  |
| **17.** | | | Do you feel able to support your child to have healthy movement behaviours? | | |  |
|  | | | 🞏 Yes 🞏 No  **If yes, go to 17a. If no, go to 17b.** | | |  |
| **17a.** | | | **If yes,** how ?_______________________ | | |  |
| **17b.** | | | **If no,** why­­­­­­­­­­­­­­­­­­? ______________________ | | |  |
| **18.** | | | In what type of housing do you and your child live? | | |  |
|  | | | Low rise apartment/condominium  High rise apartment/condominium  Townhouse  Semi-detached house  Detached house  Attached tube house with/without yards  Other (please specify): ……………………………………………….. | | |  |
| **19.** | | | How many children (under 18 years old), do you have living in this household **during COVID-19 restrictions**? | | |  |
|  | | | 1  2  3  4  5  6  7  8  9  10  More than 10 | | |  |
| **20.** | | | How many adults (18 years old or older, including yourself), do you have living in this household **during COVID-19 restrictions**? | | |  |
|  | | | 1  2  3  4  5  6  7  8  9  10  More than 10 | | |  |
| **21.** | | | What are the current working arrangements for your family? (tick all that apply) | | |  |
|  | | | Mother working at home  Father working at home  Mother going to work as normal  Father going to work as normal  Neither mother or father working  Other (please specify): …………………….. | | |  |
| **22.** | | | During a typical week under COVID-19 restrictions, how **stressed** did you feel compared to before the restrictions? | | |  |
|  | | | Less stressed 🞏 About the same 🞏 More stressed | | |  |
| **23.** | | | During a typical week under COVID-19 restrictions, how **exhausted** did you feel compared to before the restrictions? | | |  |
|  | | | Less exhausted 🞏 About the same 🞏 More exhausted | | |  |
| **24.** | | | What kind of support have you received from the childcare centre **during COVID-19 restrictions**?? (tick all that apply) | | |  |
|  | | | 🞏 A message on prevention of COVID-19 🞏 Story time via audio or video 🞏 Games, for example, structured physical activity games 🞏 Other (please specify) ……………………… 🞏 None | | |  |
| **25.** | | | What existing resources have you used to support/facilitate/monitor your child’s physical activity and/or screen time at home **during COVID-19 restrictions**?? | |  |  |
|  | | | 🞏 Smart phone/iPad/similar device apps   - list all the apps that you have used: __________________________________________________________________________________________________________________________________________________________   🞏 Television 🞏 Internet 🞏 Radio 🞏 Other ……………………… 🞏 None | |  |  |
| **26.** | | | Other comments:  ____________________________________________________________________________________________________________________________________________________________________________________________________________________________________________________________  ____________________________________________________________________________________ | |  |  |
| **27.** | | | Date survey was completed:  DD/MM/YYYY ______/________/____________ | |  |  |

**Table S2. Descriptive characteristics of participating countries**

| **Country** | **WHO region** | **Human Development Index^a^** | **World Bank classification^b^** | **Local institution and location** | **Region where data collection occurred** |
| --- | --- | --- | --- | --- | --- |
| Australia | WPRO | 0.938 (Very High) | High-income | Early Start, University of Wollongong, Australia | Urban: Wollongong and Sydney  Rural: South Coast NSW |
| Bangladesh | SEARO | 0.614 (Medium) | Lower-middle income | Biomedical Research Foundation, Dhaka, Bangladesh | Urban: Dhaka |
| China | WPRO | 0.758 (High) | Upper-middle income | Capital Institute of Pediatrics, Beijing, China | Urban: Shijinshan District (Beijing) |
| Hong Kong | WPRO | 0.939 (Very High) | High-income | The Chinese University of Hong Kong, Shatin, N.T. Hong Kong | Urban: Hong Kong Island, Kowloon & the New Territories |
| India | SEARO | 0.647 (Medium) | Lower-middle income | Kem Hospital Research Centre, Pune, India | Rural: Vadu, Shirur Taluka of Pune District |
| Indonesia | SEARO | 0.707 (High) | Lower-middle income | Universitas Pendidikan Indonesia, Jawa Barat, Indonesia | Urban: Bandung,  Rural: Ciamis |
| Malaysia | WPRO | 0.804 (Very High) | Upper-middle income | Universiti Kebangsaan Malaysia, Bangi, Selangor, Malaysia | Urban: Kuala Lumpur  Rural: Kuala Selangor |
| Morocco | EMRO | 0.676 (Medium) | Lower-middle income | Unite Mixte de Recherche en Nutrition et Alimentation, Rabat, Morocco | Urban: Rabat-Salé-kénitra Region  Rural: Rabat-Salé-kénitra Region |
| Pakistan | EMRO | 0.560 (Medium) | Lower-middle income | Precision Health Consultants Global, Karachi, Pakistan | Urban: Karachi West & Central Districts |
| Spain | EURO | 0.893 (Very High) | High-income | University of Seville, Seville, Spain | Urban: Seville and Valencia Provinces  Rural: Seville and Valencia Provinces |
| Sri Lanka | SEARO | 0.780 (High) | Upper-middle income | University of Colombo, Colombo, Sri Lanka | Urban: Colombo  Rural: Homagama |
| Sweden | EURO | 0.937 (Very High) | High-income | Karolinska Institute, Stockholm, Sweden | Urban: Stockholm County  Rural: Östergötland County |
| United States | PAHO | 0.920 (Very High) | High-income | Pennington Biomedical Research Center, Baton Rouge, Louisiana | Urban: Louisiana, South Eastern Region |
| Vietnam | WPRO | 0.693 (Medium) | Lower-middle income | Pham Ngoc Thach University of Medicine, Ho Chi Minh City, Vietnam | Urban: District Tân Bình & District 1 in Ho Chi Minh city  Rural: District Bình Chánh & District Nhà Bè in Ho Chi Minh city |

*WPRO*, Western Pacific Region; *SEARO*, South-East Asia Region; *EMRO*, Eastern Mediterranean Region; *EURO*, European Region; *PAHO*, Region of the Americas.

^a^ Obtained from the United Nations Development Programme. 2019 Human Development Index Ranking. Human Development Report Office.

**^b^** Obtained from the World Bank. Data – World Bank Country Lending Groups. 2020.

**Table S3. Characteristics of analytic sample**

| **Sample distribution** | **Total (n=948)** | **Boys (n=482)** | **Girls (n=466)** |
| --- | --- | --- | --- |
| WHO Regions |  |  |  |
| *Eastern Mediterranean Region* |  |  |  |
| Morocco | 23 | 15 | 8 |
| Pakistan | 17 | 13 | 4 |
| *European Region* |  |  |  |
| Spain | 82 | 35 | 47 |
| Sweden | 81 | 44 | 37 |
| *Region of the Americas* |  |  |  |
| United States | 21 | 11 | 10 |
| *South-East Asia Region* |  |  |  |
| Bangladesh | 39 | 21 | 18 |
| India | 47 | 21 | 26 |
| Indonesia | 81 | 38 | 43 |
| Sri Lanka | 90 | 39 | 51 |
| *Western Pacific Region* |  |  |  |
| Australia | 24 | 11 | 13 |
| China | 196 | 105 | 91 |
| Hong Kong | 64 | 36 | 28 |
| Malaysia | 104 | 53 | 51 |
| Vietnam | 79 | 40 | 39 |
| Rurality |  |  |  |
| Urban | 581 | 318 | 263 |
| Rural | 367 | 164 | 203 |
| Caregiver's highest level of education |  |  |  |
| Primary school or below | 56 |  |  |
| Secondary school | 287 |  |  |
| Tertiary education | 598 |  |  |
| Refused to answer | 7 |  |  |

**Table S4. Distribution of analytic sample according to COVID-19 factors**

| **COVID-19 factors** | **n (%)** |
| --- | --- |
| Level of restrictions  High  Moderate  Low | 385 (40.6)  437 (46.1)  126 (13.3) |
| Go outside during COVID-19  No  Yes | 158 (16.7)  790 (83.3) |
| Caregiver’s concern about child’s movement behaviour^a^ (Yes)  Physical activity  Sitting (including screen time)  Sleep | 497 (52.4)  563 (59.4)  448 (47.3) |
| Caregiver’s perceived ability to support child to have healthy movement behaviours  No  Yes | 181 (19.1)  765 (80.9) |
| Presence of outdoor space within house compound  No  Yes | 389 (41.3)  553 (58.7) |
| Number of adults living within the same household  Two or less  More than two | 612 (64.6)  336 (35.4) |
| Number of children living within the same household  Two or less  More than two | 749 (79.0)  199 (21.0) |
| Caregiver’s perceived level of stress compared to before COVID-19  Less/about the same  More stressed | 628 (66.2)  320 (33.8) |
| Caregiver’s perceived level of exhaustion compared to before COVID-19  Less/about the same  More exhausted | 657 (69.3)  291 (30.7) |
| Receiving any support from childcare centre  No  Yes | 170 (17.9)  778 (82.1) |
| Using any resources to support/facilitate child’s movement behaviours at home  No  Yes | 363 (38.3)  585 (61.7) |
| Unable to be active at home during survey period due to sickness | 46 (4.9) |
